# Platelet Activating Factor Receptor and Intercellular Adhesion Molecule–1 Expression Increases in the Small Airway Epithelium and Parenchyma of Patients with Idiopathic Pulmonary Fibrosis: Implications for Microbial Pathogenesis

**DOI:** 10.3390/jcm13072126

**Published:** 2024-04-06

**Authors:** Affan Mahmood Shahzad, Wenying Lu, Surajit Dey, Prem Bhattarai, Archana Vijay Gaikwad, Jade Jaffar, Glen Westall, Darren Sutherland, Gurpreet Kaur Singhera, Tillie-Louise Hackett, Mathew Suji Eapen, Sukhwinder Singh Sohal

**Affiliations:** 1 Respiratory Translational Research Group, Department of Laboratory Medicine, School of Health Sciences, College of Health and Medicine, University of Tasmania, Launceston, TAS 7248, Australia; 2 Medical School, Oceania University of Medicine, Apia WS1330, Samoa; 3 National Health and Medical Research Council (NHMRC) Centre of Research Excellence (CRE) in Pulmonary Fibrosis, Respiratory Medicine and Sleep Unit, Royal Prince Alfred Hospital, Camperdown, NSW 2050, Australia; 4 Department of Allergy, Immunology and Respiratory Medicine, The Alfred Hospital, Melbourne, VIC 3004, Australia; 5 Department of Immunology and Pathology, Monash University, Melbourne, VIC 3800, Australia; 6 Department of Anaesthesiology, Pharmacology and Therapeutics, University of British Columbia, Vancouver, BC V6T 1Z4, Canada; 7 Centre for Heart Lung Innovation, St. Paul’s Hospital, Vancouver, BC V6Z 1Y6, Canada

**Keywords:** alveolar macrophages, ICAM–1, idiopathic pulmonary fibrosis, infections, PAFR, type 2 pneumocytes

## Abstract

**Background**: Idiopathic pulmonary fibrosis (IPF) is an irreversible lung fibrotic disorder of unknown cause. It has been reported that bacterial and viral co-infections exacerbate disease pathogenesis. These pathogens use adhesion molecules such as platelet activating factor receptor (PAFR) and intercellular adhesion molecule-1 (ICAM–1) to gain cellular entry, causing infections. **Methods**: Immunohistochemical staining was carried out for lung resections from IPF patients (n = 11) and normal controls (n = 12). The quantification of PAFR and ICAM–1 expression is presented as a percentage in the small airway epithelium. Also, type 2 pneumocytes and alveolar macrophages were counted as cells per mm^2^ of the parenchymal area and presented as a percentage. All image analysis was done using Image Pro Plus 7.0 software. **Results**: PAFR expression significantly increased in the small airway epithelium (*p* < 0.0001), type 2 pneumocytes (*p* < 0.0001) and alveolar macrophages (*p* < 0.0001) compared to normal controls. Similar trend was observed for ICAM–1 expression in the small airway epithelium (*p* < 0.0001), type 2 pneumocytes (*p* < 0.0001) and alveolar macrophages (*p* < 0.0001) compared to normal controls. Furthermore, the proportion of positively expressed type 2 pneumocytes and alveolar macrophages was higher in IPF than in normal control. **Conclusions**: This is the first study to show PAFR and ICAM–1 expression in small airway epithelium, type 2 pneumocytes and alveolar macrophages in IPF. These findings could help intervene microbial impact and facilitate management of disease pathogenesis.

## 1. Introduction

Idiopathic pulmonary fibrosis (IPF) is a rare, chronic interstitial lung disease (ILD) associated with irreversible lung fibrosis with an unknown cause [1,2,3]. IPF has a high mortality rate with a median survival of 2–5 years from diagnosis [1,2,4]. It is commonly seen in the older demographic, particularly in individuals aged 50 and above [5,6]. Some typical clinical characteristics are chronic cough, chronic exertional dyspnea, digital clubbing, and bibasilar inspiratory crackles [7,8]. Although unknown etiology, infectious agents, genetics, tobacco, and environmental factors are related to IPF pathogenesis [1,5,9]. When irritants persist, the dysregulated repair mechanisms contribute to excess collagen deposition and fibrotic scar tissue formation [10,11,12]. As a result, the common histopathological feature of IPF is fibrosis, including the characteristic honeycombing appearance [1,13,14].

Studies over the years have discovered that IPF patients suffer from respiratory-associated infections that contribute to worsened disease pathogenesis [15,16,17]. It has been reported that IPF patients have a higher bacterial load in bronchoalveolar lavage (BAL) fluid than healthy or chronic obstructive pulmonary disease (COPD) cohorts [15,16]. The most common bacterial genus in IPF is *Streptococcus*, *Hemophilus*, *Pseudomonas*, *Neisseria*, and *Veillonella* [15,16]. Moreover, research has also found that some viruses, namely the human rhinovirus (HRV) and influenza virus, are involved in affecting patients [15,18]. A synergy between bacteria and virus has been documented, detailing that it causes superinfections that exacerbate IPF progression, reducing lung function and lowering the survival rate compared to uninfected patients [15]. Similarly, SARS-CoV-2 receptor angiotensin-converting enzyme 2 (ACE2) has been shown to upregulate in patients with IPF, increasing their susceptibility to COVID-19 [15,19]. With this knowledge, we wanted to investigate the presence of other potential microbial receptors, such as platelet-activating factor receptor (PAFR) and intracellular adhesion molecule–1 (ICAM–1), and their possible involvement in microbial entry into host cells [20,21,22,23]. An increase in such microbial receptors is bound to make the patients susceptible to infections that further drive unwarranted inflammation and remodeling changes [24,25].

Platelet-activating factor (PAF) functions as a known mediator which causes platelet aggregation, blood vessel dilation, inflammation, allergic reactions, and shock [26,27]. PAF is produced by many cells, such as platelets, endothelial cells, fibroblasts, macrophages, monocytes, mast cells, and some immune cells, such as neutrophils and eosinophils [26,27]. PAF is mainly involved in inflammatory responses [26]. Under normal conditions, PAF binds to PAFR, a G-protein-coupled seven-transmembrane receptor that activates several pathways and synthesis of some inflammatory mediators such as prostaglandins and cytokines [e.g., tumour necrosis factor alpha (TNF-alpha) and interleukin 8 (IL–8)] [26,28]. However, in terms of bacterial involvement, a study on COPD found that upregulated PAFR in epithelium helps *Streptococcus pneumoniae* to interact and colonize epithelial cells with the help of phosphorylcholine (ChoP) [23,29,30]. The ChoP is a molecular mimic of PAF found in *S. pneumoniae* walls and on some strains of *Hemophilus influenzae*, *Pseudomonas aeruginosa*, and *Acinetobacter baumannii* [23,31]. As a result, bacteria with ChoP binds to PAFR, outcompeting PAF (natural ligand), leading to bacterial transmigration across the host cells [30].

ICAM–1is a cell surface glycoprotein naturally expressed in low amounts in various cells, including immune, endothelial, and epithelial cells [32,33]. ICAM–1is overexpressed during an inflammatory response and cytokine upregulation [e.g., interferon beta (IFN-β), IL–8] [32,33]. The receptor has several roles, primarily regulating leukocyte movement and adhesion with blood vessels and inflammation [33,34]. It is expressed in inflammatory macrophages tasked with phagocytosis [33,34]. However, recent research showed that some viruses, such as HRV, use ICAM–1as a receptor to release their RNA in the host cells of patients [33,35,36]. The uncoating of viral RNA leads to genome replication in the epithelium, ultimately causing infection and exacerbating IPF pathogenesis [16,37,38].

In COPD, we have previously reported significantly increased PAFR expression in small airways and lung parenchyma, especially in smokers, compared to normal tissue [39]. It indicates that irritants causing inflammation upregulates microbial receptors in sites such as small airways and lung parenchyma, enabling microbial attachment [26,32]. We found similar changes for ICAM–1too in smokers and patients with COPD [36]. Therefore, we wanted to explore if this activity is evident in IPF and potentially provide a better understanding of microbial implications in this disease.

We hypothesize that PAFR and ICAM–1 expression is upregulated in IPF, which act as adhesion sites for bacteria/viruses, increasing the vulnerability to infection in IPF patients. This study aims to determine the expression of PAFR and ICAM–1in small airways and lung parenchymal areas mainly in type 2 pneumocytes and alveolar macrophages of patients with IPF compared to normal lungs to explain if IPF patients are more susceptible to infections.

## 2. Materials and Methods

### 2.1. Patient Demographics

Surgically resected human lung tissue from informed consent patients with end-stage IPF (n = 11) were obtained from Alfred Health (Ethics approval ID: 336-13) at the time of lung transplantation. Normal controls (NC, n = 12) were provided by James Hogg Lung Registry (Ethics approval ID: H00-50110). The NC lung tissues were from patients who had died of a cause other than respiratory disease (Table 1).

### 2.2. Immunohistochemical Staining

Paraffin-embedded lung tissue was cut to 3 µm. Tissues were dewaxed in the following reagents in order of two changes of xylene, two changes of absolute ethanol, 70% ethanol (*v*/*v*) in deionized water. These tissue sections were treated with target antigen retrieval (pH 6) in a Decloaking Chamber™ (Biocare Medical, Melbourne, VIC, Australia) at 110 °C for 15 min, followed by 3% hydrogen peroxide (*v*/*v*) (H1009, Sigma-Aldrich, Bayswater, VIC, Australia) in deionized water for 18 to 20 min. A protein block solution was applied for 5 min to the tissues before applying PAFR primary antibody. The tissue sections were immunohistochemically stained using primary antibodies: PAFR monoclonal antibody (1:50, 160,600, Cayman Chemical Company, Redfern, NSW, Australia) and ICAM–1 monoclonal antibody (1:100, MA5407, Invitrogen, Melbourne, VIC, Australia). Negative control antibody: mouse IgG1 monoclonal antibody (PAFR 1:50 and ICAM–1 1:100 dilution; X0931, Agilent Technologies, Mulgrave, VIC, Australia) incubated in an IHC humidity chamber for 90 min at ambient temperature, followed by peroxidase-conjugated polymer backbone-carried secondary antibodies for 30 min and visualized by 3-3′-diaminobenzidine (DAB) staining for 10 min (EnVisionTM Detection SystemsTM, K5007, Dako, Mulgrave, VIC, Australia), and hematoxylin stain was applied for nuclear staining.

### 2.3. Small Airway and Lung Parenchyma Quantification

Images were taken using a Leica DM500 microscope and Leica ICC50W camera (Leica, Macquarie Park, NSW, Australia), and analysis was performed using Image Pro Plus 7.0 software (Media Cybernetics, Rockville, MD, USA). The observer was blinded to patients and diagnosis. Small airway epithelium and lung parenchyma areas were captured at 40× and 20× magnification, respectively, and strictly avoiding any overlapping images. Then, eight images were randomly selected using online random generator software for measurement. The quantification of PAFR and ICAM–1 expression on the small airway epithelium is presented as a percentage of the epithelial layer. Also, type 2 pneumocytes and alveolar macrophages were counted as cells per mm^2^ of the parenchymal area and presented as a percentage.

### 2.4. Statistical Analysis

Following normality check, non-parametric analysis of variance were performed using the unpaired one-tailed Mann-Whitney Test; specific group differences without correction for multiple comparisons were assessed using a two-way ANOVA test with Fisher’s LSD test. The statistical analysis was completed using GraphPad Prism V9.1 (GraphPad Software Inc., La Jolla, CA, USA), with a *p*-value ≤ 0.05 considered statistically significant.

## 3. Results

### 3.1. Comparison between Primary Antibody and Negative Antibody Staining on NC and IPF Lung Tissue

**Figure 1:**
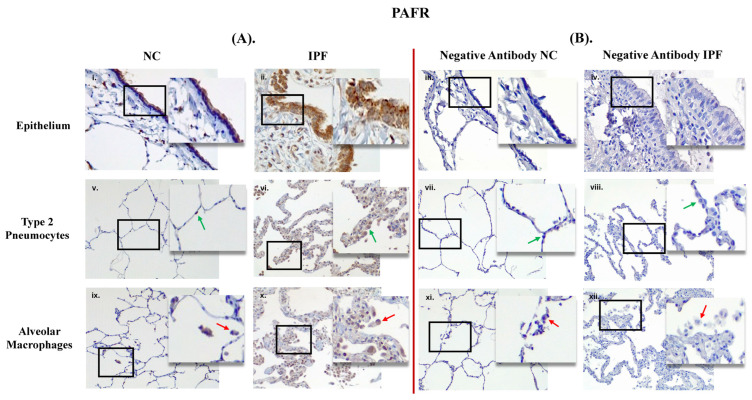
Human resected lung tissue showing positive staining (in brown) for (platelet activating factor reporter, PAFR) (**A**) and negative mouse IgG1 antibody staining (**B**). ((**A**) (**i**,**v**,**ix**)) PAFR positive in small airway (SA) epithelium, type 2 pneumocytes (green arrows), and alveolar macrophages (red arrows) in normal control (NC), respectively and ((**A**) (**ii**,**vi**,**x**)) PAFR positive in SA epithelium, type 2 pneumocytes (green arrows), and alveolar macrophages (red arrows) in patients with idiopathic pulmonary fibrosis (IPF), respectively. ((**B**) (**iii**,**vii**,**xi**)) negative IgG1 in SA epithelium, type 2 pneumocytes (green arrows), and alveolar macrophages (red arrows) in NC, respectively and ((**B**) (**iv**,**viii**,**xii**)) negative IgG1 in SA epithelium, type 2 pneumocytes (green arrows), and alveolar macrophages (red arrows) in patients with IPF, respectively. Magnification: SA epithelium (40×), lung parenchyma (20×) and insert image (60×).

**Figure 2:**
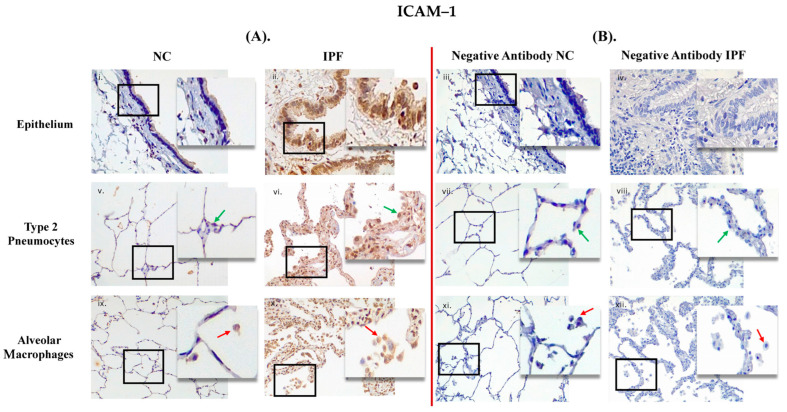
Human resected lung tissue showing positive staining (in brown) for (intercellular adhesion molecule–1, ICAM–1) (**A**) and negative mouse IgG1 antibody staining (**B**). ((**A**) (**i**,**v**,**ix**)) ICAM–1 positive in small airway (SA) epithelium, type 2 pneumocytes (green arrows), and alveolar macrophages (red arrows) in normal control (NC), respectively and ((**A**) (**ii**,**vi**,**x**)) ICAM–1positive in SA epithelium, type 2 pneumocytes (green arrows), and alveolar macrophages (red arrows) in patients with idiopathic pulmonary fibrosis (IPF), respectively. ((**B**) (**iii**,**vii**,**xi**)) negative IgG1 in SA epithelium, type 2 pneumocytes (green arrows), and alveolar macrophages (red arrows) in NC, respectively and ((**B**) (**iv**,**viii**,**xii**)) negative IgG1 in SA epithelium, type 2 pneumocytes (green arrows), and alveolar macrophages (red arrows) in patients with IPF, respectively. Magnification: SA epithelium (40×), lung parenchyma (20×) and insert image (60×).

PAFR and ICAM–1 positive expression in small airways (SA) epithelium and lung parenchyma in IPF and NC are shown in Figure 1A and Figure 2B. PAFR and ICAM–1 were showing strong positive expression in epithelium (in brown) in IPF compared to NC, and similarly more positive expression in type 2 pneumocytes and alveolar macrophages in IPF than in NC. Negative control staining is shown in Figure 1B and Figure 2B, which indicate that there is no false staining.

### 3.2. Quantification of PAFR Expression in Small Airway (SA) Epithelium, Type 2 Pneumocytes and Alveolar Macrophages

**Figure 3:**
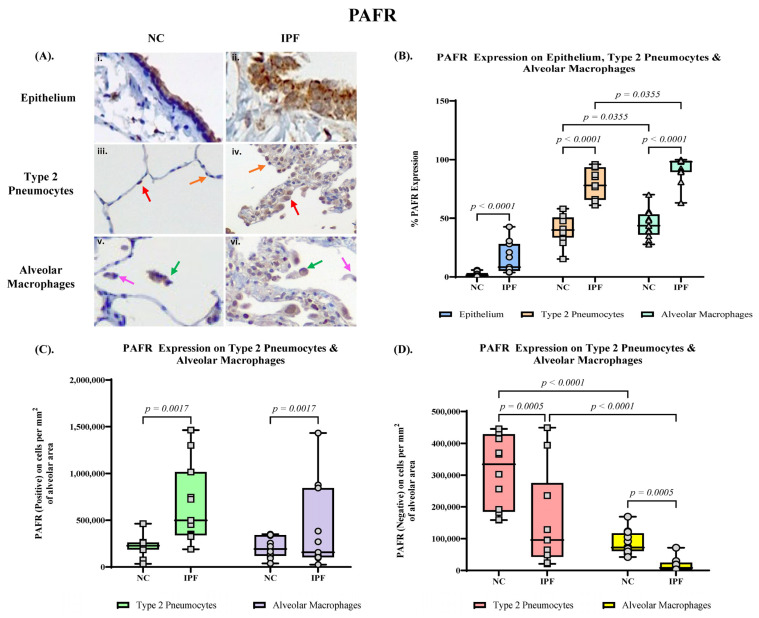
(**A**) Immunohistochemical (IHC) staining with primary antibody PAFR of normal control (NC) and idiopathic pulmonary fibrosis (IPF) tissue. The IHC staining compares ((**A**) (**i**,**ii**)) small airway (SA) epithelium in NC and IPF respectively, ((**A**) (**iii**,**iv**)) type 2 pneumocytes (positive—red arrows, negative—orange arrows) in NC and IPF respectively, and ((**A**) (**v**,**vi**)) alveolar macrophages (positive—green arrows, negative—bright pink arrows) in NC and IPF respectively. (**B**) Percentage expression of PAFR in epithelium, type 2 pneumocytes, and alveolar macrophages. Elevated SA epithelium PAFR expression (*p* < 0.0001) in IPF compared to NC. Elevated PAFR expression in type 2 pneumocytes (*p* < 0.0001) and alveolar macrophages (*p* < 0.0001) in IPF. (**C**) The number of PAFR positively expressed type 2 pneumocytes (*p* = 0.0017) and alveolar macrophages (*p* = 0.0017) per mm^2^ of alveolar area. (**D**) The number of PAFR negative type 2 pneumocytes (*p* = 0.0005) and alveolar macrophages (*p* = 0.0005) per mm^2^ of alveolar area. Magnification: SA epithelium and lung parenchyma (60×).

PAFR expression was prominent on the apical surface of small airway epithelial cells in IPF tissue, and mildly covering the whole perimeter of the cells (Figure 3A). There was negligible PAFR staining on the normal controls with patterns illustrated (Figure 3A). We further observed the expression of PAFR in the lung parenchyma, which increased mainly in type 2 pneumocytes and alveolar macrophages in IPF tissue compared to NC tissue (Figure 3A).

The percentage expression of PAFR significantly upregulated in the small airway epithelium in IPF lung tissue (median 8.33%, range 3.80–42.7%) compared to NC lung tissue (median 1.43%, range 0.352–5.85, *p* < 0.0001) (Figure 3B). In addition, the PAFR positive expression in alveolar type 2 pneumocytes of IPF showed a significantly high percentage (median 78.1%, range 61.2–96.0%) compared to NC (median 40.1%, range 15.2–58.2%, *p* < 0.0001) (Figure 3B). The number of PAFR positive type 2 pneumocytes per mm^2^ in alveolar area is significantly higher in IPF (median 498,525 cells per mm^2^, range 189,449–1,463,564 cells per mm^2^) compared to NC (median 227,150 cells per mm^2^, range 34,483–463,444 cells per mm^2^, *p* = 0.0017) (Figure 3C), and in contrast, the number of PAFR negative type 2 pneumocytes is significantly higher in NC (median 334,189 cells per mm^2^, range 158,333–445,101 cells per mm^2^) compared to IPF (median 96,519 cells per mm^2^, range 20,930–825,189 cells per mm^2^, *p* = 0.0017) (Figure 3D).

We also observed significant upregulation of PAFR expression in alveolar macrophages (median 98.3%, range 63.2–100%) compared to NC (median 43.7%, range 27.8–70.2%, *p* < 0.0001) (Figure 3B). Similarly, the number of PAFR positive alveolar macrophages is significantly higher in IPF (median 157,029 cells per mm^2^, range 25,707–1,432,457 cells per mm^2^) compared to NC (median 74,713 cells per mm^2^, range 22,630–150,741 cells per mm^2^, *p* = 0.0005) (Figure 3C). The number of PAFR negative alveolar macrophages is significantly higher in NC (median 72,072 cells per mm^2^, range 42,026–169,141 cells per mm^2^) compared to IPF (median 8048 cells per mm^2^, range 0–71,342 cells per mm^2^, *p* = 0.0005) (Figure 3D).

### 3.3. Quantification of ICAM–1 Expression in Small Airway (SA) Epithelium, Type 2 Pneumocytes and Alveolar Macrophages

**Figure 4:**
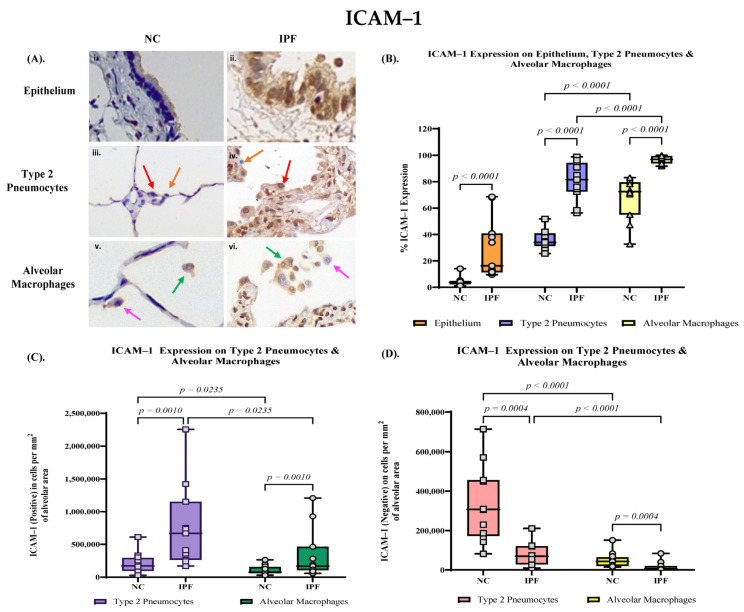
(**A**) Immunohistochemical (IHC) staining with primary antibody ICAM–1 of normal control (NC) and idiopathic pulmonary fibrosis (IPF) tissue. The IHC staining compares: ((**A**) (**i**,**ii**)) small airway (SA) epithelium in NC and IPF respectively, ((**A**) (**iii**,**iv**)) type 2 pneumocytes (positive—red arrows, negative—orange arrows) in NC and IPF respectively, and ((**A**) (**v**,**vi**)) alveolar macrophages (positive—green arrows, negative—bright pink arrows) in NC and IPF respectively. (**B**) Percentage expression of ICAM–1 in epithelium, type 2 pneumocytes, and alveolar macrophages. Elevated SA epithelium ICAM–1 expression (*p* < 0.0001) in IPF compared to NC. Elevated ICAM–1 expression in type 2 pneumocytes (*p* < 0.0001) and alveolar macrophages (*p* < 0.0001) in IPF. (**C**) The number of ICAM–1 positively expressed type 2 pneumocytes (*p* = 0.0010) and alveolar macrophages (*p* = 0.0010) per mm^2^ of alveolar area. (**D**) The number of ICAM–1 negative type 2 pneumocytes (*p* = 0.0004) and alveolar macrophages (*p* = 0.0004) per mm^2^ of alveolar area. Magnification: SA epithelium and lung parenchyma (60×).

ICAM–1 expression was prominent in the nucleus of small airway epithelial cells in IPF tissue, and moderate in cytoplasm (Figure 4A). There was mild ICAM–1 staining on the normal controls with patterns illustrated (Figure 4A).

The percentage expression of ICAM–1 significantly upregulated in the small airway epithelium in IPF lung tissue (median 16.3%, range 9.56% to 68.5%) compared to NC lung tissue (median 3.76%, range 1.17–14.1%, *p* < 0.0001) (Figure 4B). Compared to NC lung tissue (median 34.0%, range 25.6–51.7%), the ICAM–1 positive expression in type 2 pneumocytes of IPF alveolar tissue showed a significantly high percentage of type 2 cells in alveolar area (median 81.5%, range 56.3–98.8%, *p* < 0.0001) (Figure 4B). The number of ICAM–1 positive type 2 pneumocytes per mm^2^ in alveolar area is significantly higher in IPF (median 668,143 cells per mm^2^, range 168,350–2,253,902 cells per mm^2^) compared to NC (median 169,437 cells per mm^2^, range 28,250–611,925 cells per mm^2^, *p* = 0.0010) (Figure 4C), and in contrast, the number of ICAM–1 negative type 2 pneumocytes is significantly higher in NC (median 307,286 cells per mm^2^, range 82,058–713,380 cells per mm^2^) compared to IPF (median 69,772 cells per mm^2^, range 8858–210,993 cells per mm^2^, *p* = 0.0010) (Figure 4D).

We also observed significant upregulation of ICAM–1 expression in alveolar macrophages (median 96.8%, range 91.7–100%) compared to NC (median 72.5%, range 32.7–83.1%, *p* < 0.0001) (Figure 4B). The number of ICAM–1 positively expressed alveolar macrophages is significantly higher in IPF (median 167,813 cells per mm^2^, range 56,157–1,209,594 cells per mm^2^) compared to NC (median 71,714 cells per mm^2^, range 29,595–56,157 cells per mm^2^, *p* = 0.0004) (Figure 4C). The number of ICAM–1 negative alveolar macrophages is significantly higher in NC (median 42,969 cells per mm^2^, range 14,497–150,936 cells per mm^2^) compared to IPF (median 2891 cells per mm^2^, range 0–39,443 cells per mm^2^, *p* = 0.0004) (Figure 4D).

### 3.4. Proportion of PAFR and ICAM–1 Expression in IPF and NC in the Alveolar Area

**Figure 5:**
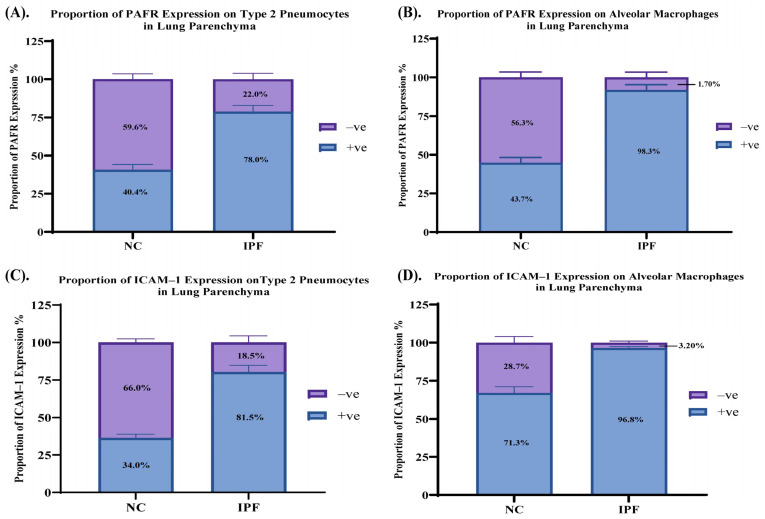
Proportion of PAFR and ICAM–1 expression in type 2 pneumocytes and alveolar macrophages in lung parenchyma. Elevated PAFR expression in IPF, (**A**) type 2 pneumocytes and (**B**) alveolar macrophages. Elevated ICAM–1 expression in IPF, (**C**) type 2 pneumocytes and (**D**) alveolar macrophages.

PAFR positive expression in type 2 pneumocytes in IPF elevated to 78.0% compared to 40.4% in NC. On the other hand, PAFR negative expression in type 2 pneumocytes in IPF (22.0%) was lower than in NC (59.6%) (Figure 5A). Similarly, PAFR positive expression in alveolar macrophages was higher in IPF (98.3%) compared to in NC (43.7%), whereas negative PAFR expression in IPF (1.70%) was lower than in NC (56.3%) (Figure 5B). ICAM–1 positive expression in type 2 pneumocytes in IPF elevated to 81.5% compared to 34.0% in NC. On the other hand, ICAM–1 negative in type 2 pneumocytes in IPF (18.5%) was lower than in NC (66.0%) (Figure 5C). Similarly, ICAM–1 positive expression in alveolar macrophages was higher in IPF (96.8%) compared to in NC (71.3%), whereas negative ICAM–1 expression in IPF (3.20%) was lower than in NC (28.7%) (Figure 5D).

## 4. Discussion

This is the first study reporting PAFR and ICAM–1 adhesion molecules in the small airway epithelium and lung parenchyma of patients with IPF. We found that PAFR and ICAM–1 expression increased in the small airway epithelium in IPF compared to normal tissues. In the lung parenchyma of IPF patients, we found elevated levels of PAFR and ICAM–1 positive type 2 pneumocytes and alveolar macrophages. Furthermore, the proportions of positively and negatively expressed cells showed more positively expressed type 2 pneumocytes and alveolar macrophages in IPF and fewer negatively expressed cells in normal tissue. It could imply that although alveolar macrophages innately recognize pathogens but, cell activation and molecular receptor expression may provide shelter to microbes against inflammatory molecules, contributing to increased infection [39] The above results suggest that high molecule expression in IPF could be a significant link to microbes ‘anchoring’ and gaining cellular entry, increasing the risk of infection. As IPF is a chronic pulmonary disease, postulated risk factors involved in disease pathogenesis include environmental factors, tobacco, genetics, and infectious agents [1,5]. These irritants result in a chronic injury, and inflammation affecting the molecular and cellular mechanisms [3,40]. In chronic disease, microbes infiltrate the exacerbated inflammation and fibroblast hyperproliferation, leading to excess collagen deposition [40,41].

**Figure 6:**
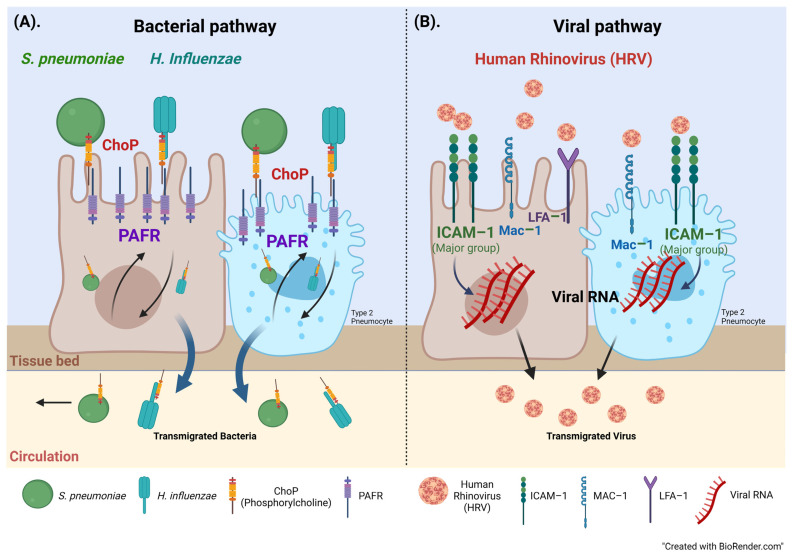
(**A**) *S. pneumoniae* and *H. influenzae* express phosphorylcholine (Chop) which binds to platelet acting factor receptor (PAFR), facilitating the adhesion, colonization and eventual transmigration into the human airway epithelial cells and type 2 pneumocytes, increasing the risk of infection in patients. (**B**) Human Rhinovirus (HRV) uses intercellular adhesion molecule–1 (ICAM–1) as a receptor to anchor onto the airway epithelial cells and type 2 pneumocytes followed by uncoating of cell-invading virus, worsening disease pathogenesis. MAC–1; macrophage–1 antigen and LFA–1; leukocyte function-associated antigen.

Phosphorylcholine (ChoP, a molecular mimic of PAF) is an important element expressed on the outer surface of various microorganisms; the most commonly seen bacterial genus in the respiratory tract is *S. pneumoniae* [42,43], *H. influenzae* [44] and *P. aeruginosa* and *A. baumannii*, which can direct interaction with host cells through ChoP [23,29,39,45]. The attachment of bacteria that express ChoP to the PAFR facilitates their adhesion to and invasion into human cells [46,47]. The general mechanism of chronic bacterial colonization has been well documented in COPD but under-researched in IPF [39]. In our study, with increased expression of PAFR in small airways indicates that these bacterial pathogens can adhere to small airway epithelium because the ChoP outcompetes the PAF (natural ligand to PAFR) and anchoring to PAFR may increase the risk of infection [15,16] (Figure 6A).

HRVs contribute to over 50% of upper respiratory tract infections [48], and HRV infections can lead to life-threatening effects that worsen chronic respiratory disease, such as COPD, asthma, or cystic fibrosis [49]. The major signaling pathway for HRV cellular access occurs because pathogens use ICAM–1 as a receptor [32,50]. Specifically, the pathogen binds to leukocyte function-associated antigen 1 (LFA–1) or the macrophage–1 antigen (Mac–1), natural adhesion ligands [32,50] (Figure 6B). ICAM–1 becomes a major catalyst for the eventual uncoating of the cell-invading virus, potentially exacerbating IPF pathogenesis [16,32]. The role of ICAM–1 in COPD has been investigated by our research group and we have showed that the receptor was highly expressed in COPD smoking cohort [36]. The expression patterns were prominent in the airways, especially on goblet cells and sub-mucosal glands, and could be a potential risk factor of infection by common respiratory viral and bacterial pathogens [36]. These biochemical mechanisms documented in other diseases shine a light on our findings as we believe our data bridges the gap in this area by demonstrating the abovementioned microbial activity in IPF, but this warrants further research.

Furthermore, our research group previously reported that ACE2, Furin and Transmembrane protease serine 2 (TMPRSS2) receptors are upregulated in IPF facilitating SARS–CoV–2 infection [19]. We have reported similar findings in smokers and patients with COPD. Small airway epithelium, type 2 pneumocytes and alveolar macrophages were highly positive for these markers [51]. Subsequently the expression patterns of various microbial receptors discussed above could suggest that IPF patients are at a higher risk of infections compared to healthy people.

Further, Moghoofiel et al. & Mostafaei et al. investigated bacterial coinfection in IPF and its possible role in disease progression. Their results demonstrated that coinfections (bacterial and viral) significantly exacerbate disease progression, enhancing the risk of death in IPF patients [15,16]. This investigation served potential usefulness in understanding the underlying mechanisms in IPF infections and could provide insights in future therapeutics. Similar views shared by Santos et al. that if interventions can decrease or prevent pathogen adherence to the epithelium, it could protect high-risk populations before the disease has progressed [52].

Moreover, an animal model study by Iovino et al. investigated the role of PAFR in pneumococcal disease. The study demonstrated that although the absence of the PAFR gene (*Pafr-/-*) mice had higher bacterial (*S. pneumoniae*) growth in the lungs at 24 h post-inoculation, the wild-type (WT) mice had higher bacteremia after 48 h [53]. In WT, bacteria dispersed throughout the body and the central nervous system (CNS) compared to restricted local infection in the pulmonary area of *Pafr-/-* mice [30,54].

Associated animal model research on the role of PAFR in pneumococcal pneumonia found that all WT mice died earlier after infection [53]. At the same time, the mortality rate was delayed and reduced in *Pafr*-/- mice [30,53]. These models show that *Pafr*-/- mice had a lower chance of developing an infection, especially when using PAFR antagonism [30,53]. Further, heavy inflammation was detected in WT mice lungs compared to *Pafr*-/- [53]. The results highlight that PAFR influences disease severity and could support the idea that molecule antagonisms could intervene in microbial activity, but further research in human subjects is warranted. This study’s strengths include using resected human lung tissues to show expression, providing new information on disease pathogenesis, and assisting with future treatment or management strategies. This research has a few limitations, with the main challenge being the small number of IPF disease tissues available but they are rare human IPF tissue. Future investigation should aim for large sample sizes for better distribution and correlation with measured parameters. Secondly, the age of the normal control group is relatively younger than that of the IPF group due to the limited accessibility of obtaining the tissue. Further, comparing our data with previous research was challenging as very little human clinical work is done on these receptors in IPF, hence this is a novel study in IPF. The future applications for this study include performing cell cultures to understand the cellular activity between IPF and NC. Finally, need to further investigate microbial adherence to the respiratory epithelium and inflammatory cells as well as molecular analysis to study the role of genetics and proteins in relation to disease pathogenesis and treatment.

## 5. Conclusions

In conclusion, this is the first study to show PAFR and ICAM–1 expression in small airway epithelium, type 2 pneumocytes and alveolar macrophages in IPF patients compared to normal controls. Following previous research in COPD and IPF, high expression of these adhesion molecules could bridge the gap on inflammation and microbial activity in IPF. The clinical significance of these expression patterns suggests that microbes (bacteria and viruses) use these molecules as a mode of ‘anchor’ to gain cellular entry during inflammatory response, exacerbating disease pathogenesis. These findings can potentially help with future therapeutic development that can halt the disease progression, increasing the survival rate and reducing the global burden.

## Figures and Tables

**Table 1:** Patient demographics and lung function parameters.

	**Normal Control (NC)**	**IPF**
**Factors**	Values *	
Total Number (*n*)	12	11
Age (Years)	39 ± 16.5	63 ± 4.85
Gender (Female/Male)	6/6	5/6
Smoking status (*n*): Current smoker/Ex-smoker/Never	Non-smoker	0/5/6
Smoking Packs Per Year	-	16.55 ± 22.56
**Respiratory Function Parameters**		
FEV1 (L) *	NA	1.67 ± 0.42
FVC (L) ^†^	NA	1.89 ± 0.45
DLCO ^‡^ (mL/min/mmHg)	NA	5.98 ± 3.12
DLCO (%)	NA	25.33 ± 12.30

Values * presented as mean ± standard deviation (SD); NA, not available; FEV1 *, forced expiratory volume1; FVC ^†^, forced vital capacity; DLCO ^‡^, Diffusing capacity for carbon monoxide.

## Data Availability

The data that support the findings of this study are available from the corresponding author upon reasonable request.
